# Commentary: Psychological distress among healthcare students in Poland from COVID-19 to war on Ukraine: a cross-sectional exploratory study

**DOI:** 10.3389/fpubh.2025.1529520

**Published:** 2025-01-22

**Authors:** Mateusz Guziak, Maciej Walkiewicz

**Affiliations:** ^1^Division of Quality of Life Research, Faculty of Health Sciences With the Institute of Maritime and Tropical Medicine, Medical University of Gdańsk, Gdańsk, Poland; ^2^Laboratory for Strengthening the Capacity and Performance of Health Systems and Health Workforce for Health Equity, Faculty of Medicine, University of Belgrade, Belgrade, Serbia

**Keywords:** mental health, psychological distress, COVID-19, Ukraine war, education, medical students

## Introduction

This commentary reflects on the article, “*Psychological distress among healthcare students in Poland from COVID-19 to war on Ukraine: a cross-sectional exploratory study*” (1). The authors highlight the pressing need for mental, psychological, and social support interventions to mitigate the alarming levels of anxiety, stress, and depression among healthcare students. In this response, we build on their findings by sharing recent insights from our studies on the availability and accessibility of psychological support for medical students in Poland.

## Mental health of medical students in Poland

The article rightly underscores the pervasive psychological distress exacerbated by the dual crises of the COVID-19 pandemic and the ongoing war in Ukraine (1). A substantial number of Polish medical students have reported experiencing depressive symptoms, risky alcohol consumption, burnout, and high levels of stress (2, 3). Research indicates that during the COVID-19 pandemic, 44% of medical students considered dropping out due to academic stress, while 33.6% experienced suicidal thoughts or self-harm ideation (3). Another study reveals that 39.1% of students felt the need for psychological or psychiatric support during the pandemic, and 26.4% of those with pre-existing mental health conditions saw their symptoms worsen. Additionally, 28.6% reported increased use of alcohol, cigarettes, or other stimulants (4). These alarming data highlight the urgent need for preventive, and intervention programs aimed at improving life satisfaction, enhancing subjective health assessments, and strengthening coping skills to reduce stress and anxiety (1, 5–7). The need to evaluate the effectiveness of mental health initiatives form medical students has been identified as a critical research gap. Such analyses are particularly relevant in the Polish context, where data on the outcomes of these programs remain scarce. Addressing this gap is essential to provide evidence-based recommendations for improving psychological support for medical students.

## Discussion

Despite the clear need for structured and evidence-based regulations to support student wellbeing, Poland lacks a systemic approach. In contrast, countries such as the United Kingdom have implemented regulations and guidelines that ensure the availability of mental health services for students (6). Since our first research on the availability of psychological support for medical students in Poland, the landscape of medical education has changed significantly, not only due to the unprecedented challenges posed by the COVID-19 pandemic and the ongoing war in Ukraine but also by the substantial increase in both admission quotas and the number of institutions offering medical training. In 2019, we explored several key aspects of psychological support for medical students in Poland, including financing, the issue of resistance and neutrality, whether universities truly understand students' needs, and how effectively they communicate with young people (6). By 2024, we observed a significant rise in the demand for psychological support, especially following the COVID-19 pandemic, with some universities specifically establishing dedicated programs during or after this period. However, disparities remain in how institutions assess and audit these services. Some universities do not share this information. The response rate for the study has significantly declined compared to the first edition, highlighting the necessity for further research (7).

In light of these findings, it is evident that while some progress has been made in providing psychological support to medical students in Poland, some gaps remain in ensuring comprehensive and accessible services across all institutions. Such inconsistencies highlight the urgent need for a unified, national strategy that ensures comprehensive mental health support for Polish medical students, ultimately enhancing their overall wellbeing. The absence of a systemic approach to mental health in Poland can be attributed to several interconnected factors. Financial limitations represent a critical barrier, as data from the Organization for Economic Cooperation and Development (OECD) indicate that Poland allocates only ~3% of its healthcare budget to mental health services, significantly below the recommended 5–10% in high-income countries (8, 9). This chronic underfunding restricts the development of comprehensive mental health programs and limits their accessibility to students. Bureaucratic inefficiencies further exacerbate the issue, as the fragmented structure of mental health services in Poland complicates the implementation of unified strategies (10). Additionally, Poland faces a critical shortage of mental health professionals (11, 12). The lack of trained psychologists and counselors further limits the capacity to provide timely and effective support. Addressing these challenges requires a multi-faceted approach. Increased investment in mental health services, streamlining of bureaucratic processes, and strategic efforts to train and retain mental health professionals are essential steps toward building a cohesive national strategy. Central to this approach is enhancing communication about available psychological services through university platforms to ensure that students are aware of and can access the support they need. Additionally, the development of policy initiatives to standardize mental health support systems across institutions might be helpful. These policies should include dedicated funding mechanisms to ensure consistent quality and availability of services. Collaboration between universities also needs to be prioritized to enable the sharing of best practices and resources, fostering a unified approach to addressing students' psychological needs. Without these measures, the disparities and gaps in mental health support for medical students are likely to persist, undermining their wellbeing and academic performance.
